# Supplementary figures and images for: Factors indicating intention to vaccinate with a COVID-19 vaccine among older U.S. adults
Source: PLoS One. 2021 May 24;16(5):e0251963. doi: 10.1371/journal.pone.0251963 (PMC8143399; doi:10.1371/journal.pone.0251963)

A

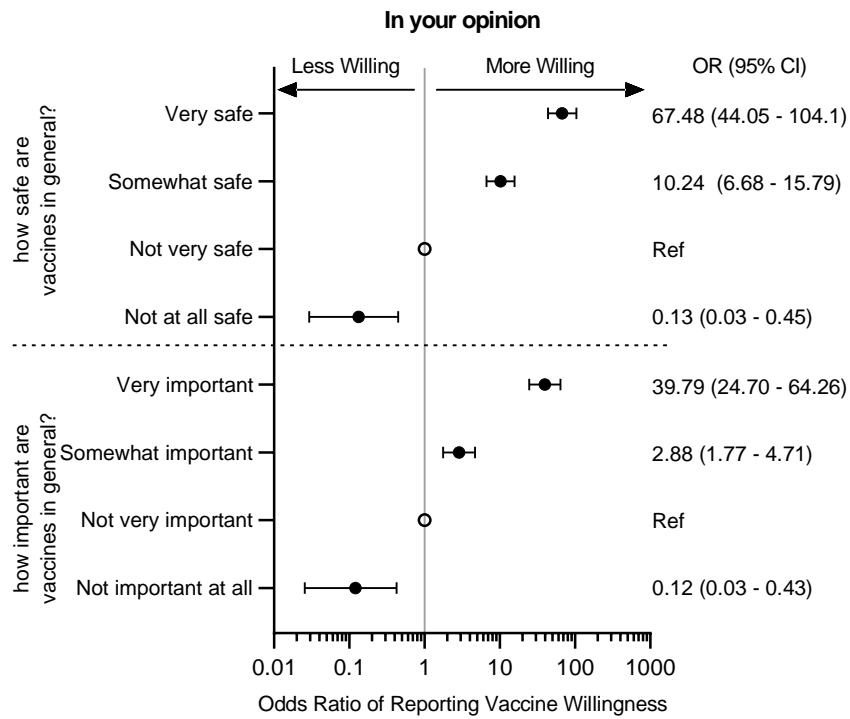

B

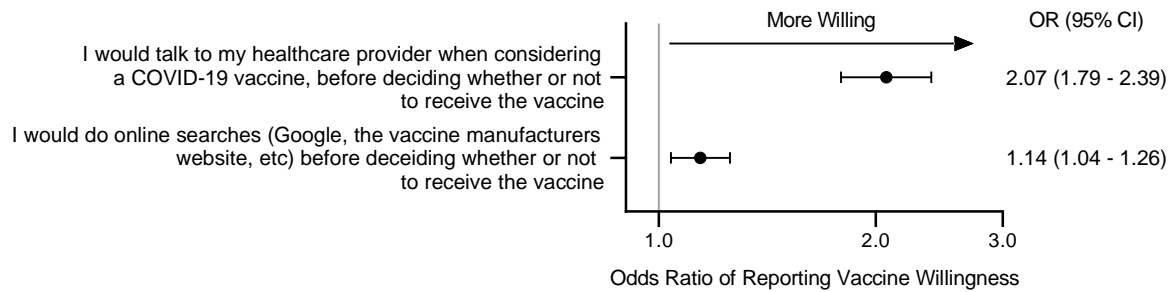

Supplement: S1 Fig — Shown are Odds Ratios (95% CI) for willingness to vaccinate. Odds Ratios were calculated using ordered logistic regression model with the 4 levels of willingness to be vaccinated as the outcome while adjusting for gender and race. (PDF) [file pone.0251963.s001.pdf]

A.

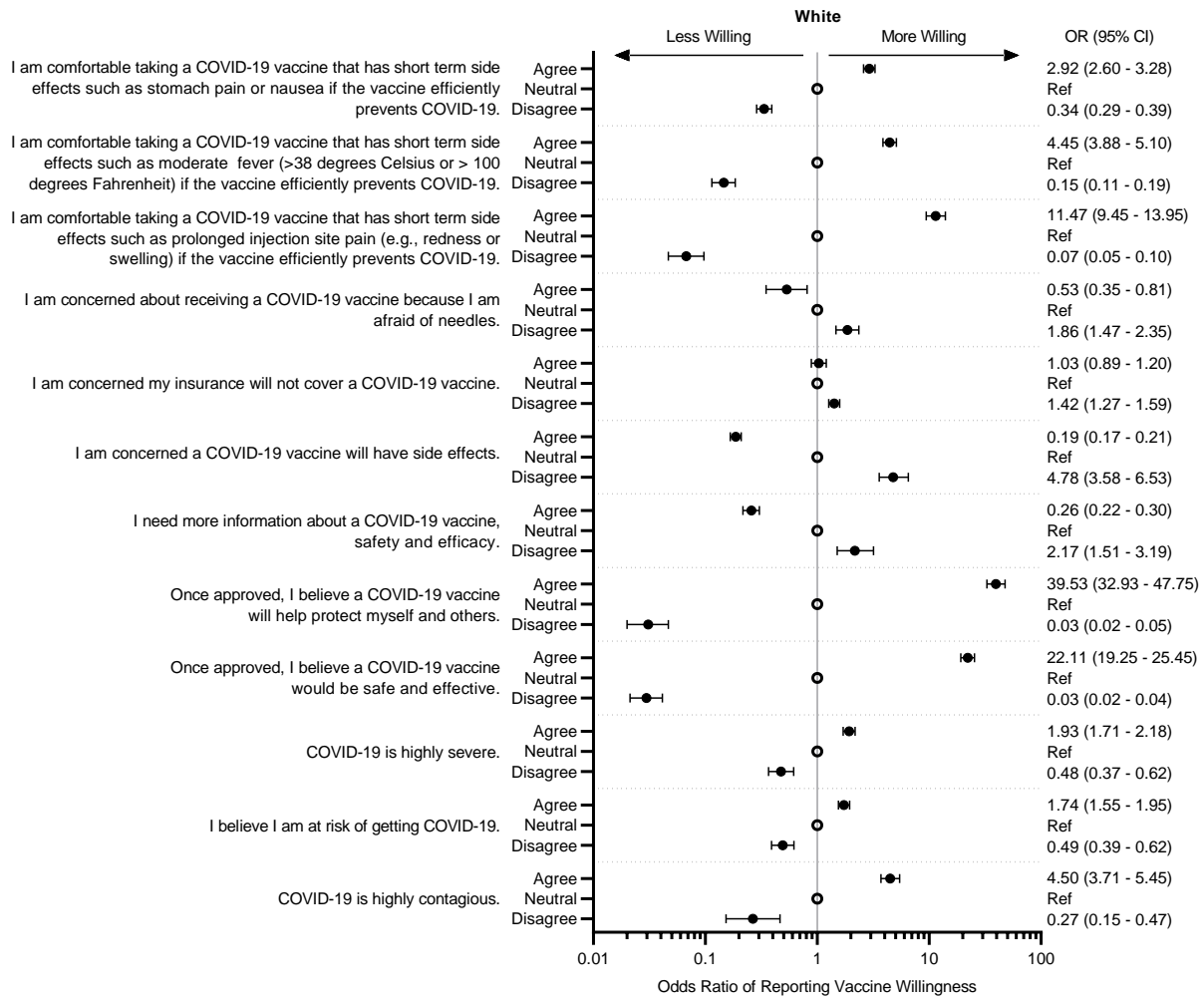

B.

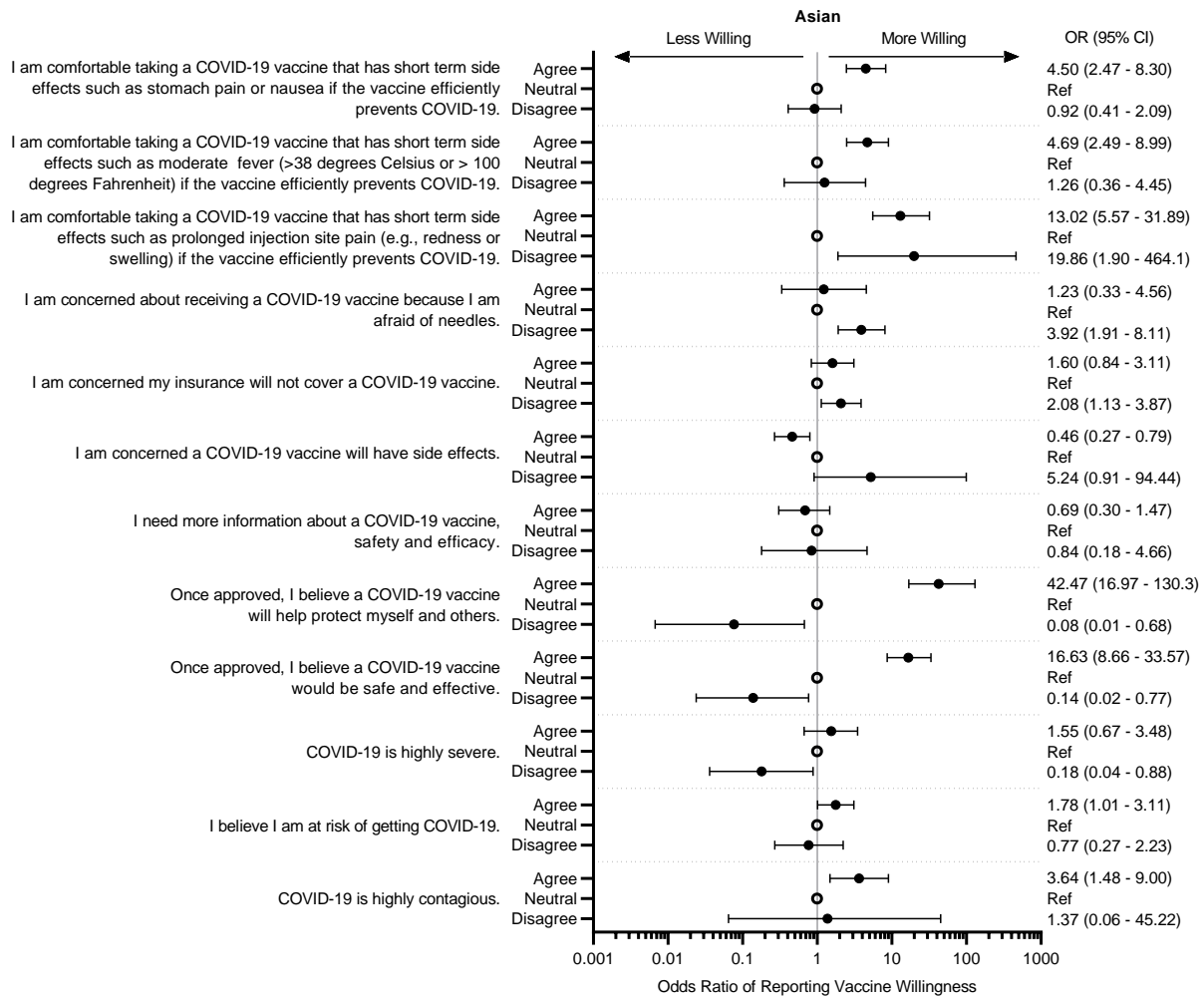

C.

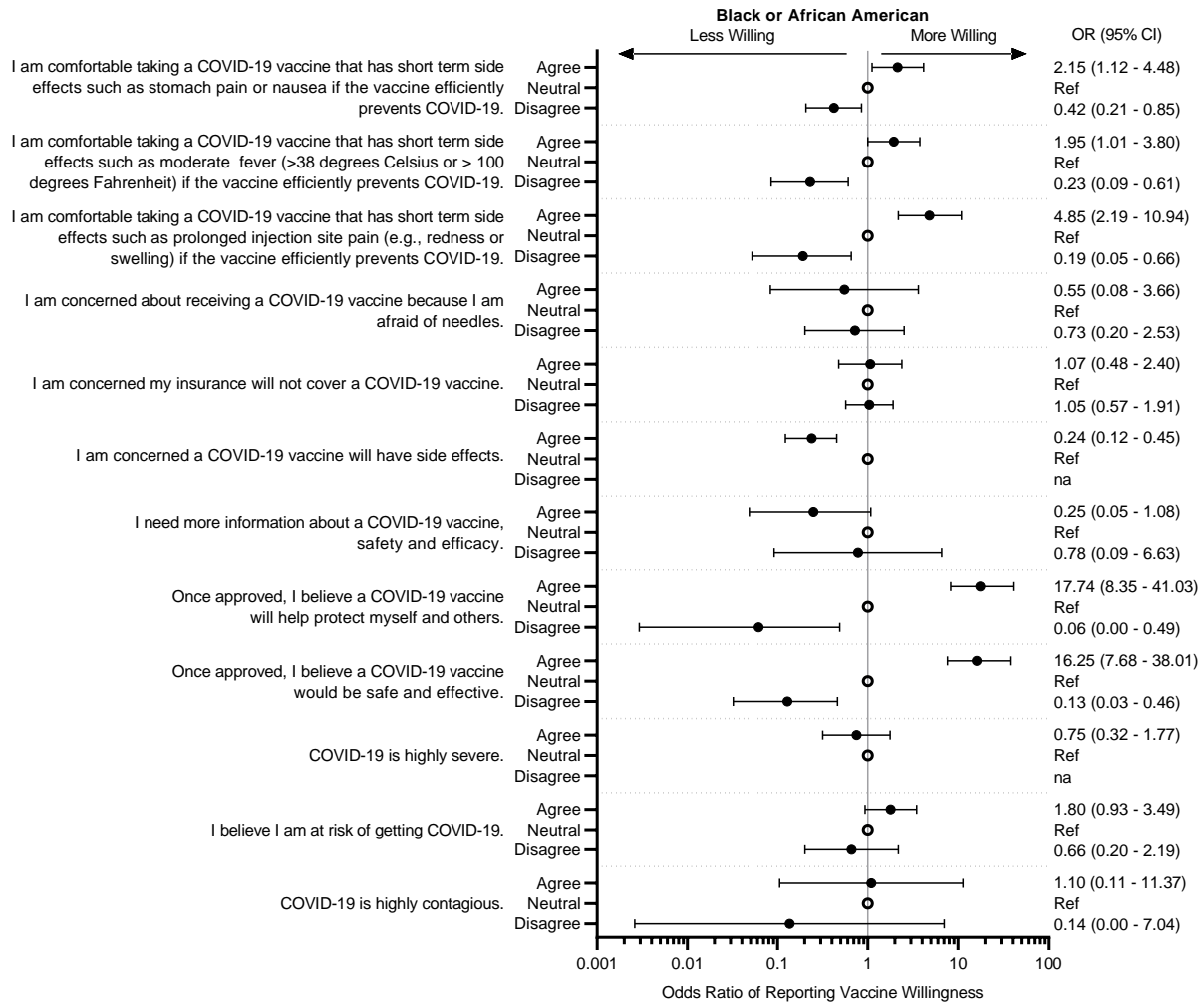

Supplement: S2 Fig — Shown are Odds Ratios (95% CI) for willingness to vaccinate for the survey by race: White (A), Asian (B), and Black (C). Odds Ratios were calculated using ordered logistic regression model with the 4 levels of willingness to be vaccinated as the outcome while adjusting for gender and race. Reference for each survey question is the option ‘neutral’ and is indicated by an open circle. na indicates insufficient subjects for this category. (PDF) [file pone.0251963.s002.pdf]
