# Supplementary material for: Factors indicating intention to vaccinate with a COVID-19 vaccine among older U.S. adults
Source: PLoS One. 2021 May 24;16(5):e0251963. doi: 10.1371/journal.pone.0251963 (PMC8143399; doi:10.1371/journal.pone.0251963)
Supplement: S1 Table — (DOCX) [file pone.0251963.s003.docx]

**Vaccine Hesitancy Assessment**

**Beliefs about vaccines**

1. **In your opinion, how important are vaccines in general?** Very important; Somewhat important; Not very important; Not important at all
2. **In your opinion, how safe are vaccines in general?** Very safe; Somewhat safe; Not very safe; Not at all safe
3. **Which of the following statements are true about your experience with vaccines in general?** *(Select all that apply)* I have not had a problem after receiving a vaccine; The side effects of vaccines are generally manageable; I am concerned about the short-term side effects; I am concerned about the long-term side effects; I have a fear of vaccines or needles; I feel that I may be putting myself at risk by receiving a vaccine; None of the above *(exclusive)*
4. **Which of the following statements are true about your knowledge of vaccines in general?** *(Select all that apply)* I believe I have enough information about vaccines; I believe they are safe and effective; I believe the vaccine approval process is highly and well regulated; I do not have enough information about vaccines; I do not believe they are safe and effective; I saw or heard negative news about vaccines; None of the above *(exclusive)*

**Willingness to be vaccinated for COVID-19**

1. **Has the COVID-19 pandemic made you more likely to vaccinate, in general, as compared to pre-pandemic times?** Much more likely; Somewhat more likely; Just as likely; Somewhat less likely; Much less likely
2. **When considering a COVID-19 vaccine, which of the following people (if any) would you talk to before deciding whether or not to receive the vaccine?** *(Select all that apply)* My pharmacist; My healthcare provider or their staff; My friends or family; My significant other or partner; Religious leaders; Community leaders; Trusted online forums or sites; None of the above *(exclusive)*
3. **Where would you look to for information to help you decide whether or not to receive a COVID-19 vaccine?** *(Select all that apply)* Online searches (Google, the vaccine manufacturers website, etc.); Clinicaltrials.gov; My healthcare provider’s office; Information from a patient support or advocacy group; Social media or other online forums ; None of the above *(exclusive)*
4. **Once available to the public, how willing would you be to receive a vaccine to protect you against COVID-19?** Very willing; Somewhat willing; Not very willing; Not at all willing
5. **If you were to receive a COVID-19 vaccine, where would you prefer to have it administered?** *(Select all that apply)* Not applicable (exclusive); Healthcare provider’s office; Hospital; Pharmacy (e.g., Walgreens, CVS, other); Workplace; Community health clinic; Religious centers; A mobile health unit (e.g., van or health ten
6. **COVID-19 is highly contagious.** Agree; Neutral; Disagree
7. **COVID-19 is highly severe**. Agree; Neutral; Disagree
8. **I believe I am at risk of getting COVID-19.** Agree; Neutral; Disagree
9. **Once approved, I believe a COVID-19 vaccine would be safe and effective.** Agree; Neutral; Disagree
10. **Once approved, I believe a COVID-19 vaccine will help protect myself and others.** Agree; Neutral; Disagree
11. **I prefer my healthcare provider recommend that I receive a COVID-19 vaccine before I receive it.** Agree; Neutral; Disagree
12. **I prefer my family or social network also receive a COVID-19 vaccine before I receive it.** Agree; Neutral; Disagree
13. **I need more information about a COVID-19 vaccine’s safety and efficacy.** Agree; Neutral; Disagree
14. **I am concerned a COVID-19 vaccine will have side effects.** Agree; Neutral; Disagree
15. **I am concerned my insurance will not cover a COVID-19 vaccine.** Agree; Neutral; Disagree
16. **I am concerned about receiving a COVID-19 vaccine because I’m afraid of needles.** Agree; Neutral; Disagree
17. **If a COVID-19 vaccine requires two doses, I believe that taking the first dose will sufficiently reduce my risk of contracting COVID-19.** Agree; Neutral; Disagree
18. **If a COVID-19 vaccine requires two doses, I believe that taking both doses will reduce my risk of contracting COVID-19.** Agree; Neutral; Disagree
19. **I am comfortable taking a COVID-19 vaccine that has short term side effects such as prolonged injection site pain (e.g., redness or swelling) if the vaccine efficiently prevents COVID-19.** Agree; Neutral; Disagree
20. **I am comfortable taking a COVID-19 vaccine that has short term side effects such as moderate fever (>38 degrees Celsius or > 100 degrees Fahrenheit) if the vaccine efficiently prevents COVID-19.** Agree; Neutral; Disagree
21. **I am comfortable taking a COVID-19 vaccine that has short term side effects such as stomach pain or nausea if the vaccine efficiently prevents COVID-19.** Agree; Neutral; Disagree
