# Supplementary material for: Factors indicating intention to vaccinate with a COVID-19 vaccine among older U.S. adults
Source: PLoS One. 2021 May 24;16(5):e0251963. doi: 10.1371/journal.pone.0251963 (PMC8143399; doi:10.1371/journal.pone.0251963)
Supplement: S2 Table — (DOCX) [file pone.0251963.s004.docx]

| **Variable** | **Levels** | **Features (n)** |
| --- | --- | --- |
| Gender | 1=Man, 0=Woman | 1 |
| Age | [65,70), [70,75), [75,80), [80,85)  [85,90), [90,95), [95,100) | 7 |
| BMI | Under weight (<18.5), Normal weight (18.5 - 25), Overweight (25.0 - 30), Obese (>= 30) | 4 |
| Race | American Indian or Alaska Native, Asian  Black or African American, Native Hawaiian or Other Pacific Islander, White, Prefer not to answer / Two or more | 6 |
| Education | Some high school or less, High school diploma or equivalent (GED), Some college education, Associate degree (e.g. AA, AS), Bachelor’s degree (e.g. BA, BS)  Master’s degree (e.g. MA, MS, MEd), Doctorate (e.g. PhD, EdD), Professional degree (e.g. MD, DDS, DVM), Prefer not to answer | 9 |
| Income | Under $30,000, $30,000-$39,999, $40,000-$49,999, $50,000-$59,999, $60,000-$74,999, $75,000-$99,999, $100,000-$149,999, $150,000-$200,000, Above $200,000, Prefer not to answer | 10 |
| In your opinion, how safe are vaccines in general? | Not at all safe, Not very safe, Somewhat safe, Very safe | 4 |
| Once available to the public, how willing would you be to receive a vaccine to protect you against COVID-19? | Not at all willing, Not very willing, Somewhat willing, Very Willing | 4 |
| COVID-19 is highly contagious. | Disagree, Neutral, Agree | 3 |
| COVID-19 is highly severe. | Disagree, Neutral, Agree | 3 |
| I believe I am at risk of getting COVID-19. | Disagree, Neutral, Agree | 3 |
| Once approved, I believe a COVID-19 vaccine would be safe and effective. | Disagree, Neutral, Agree | 3 |
| Once approved, I believe a COVID-19 vaccine will help protect myself and others. | Disagree, Neutral, Agree | 3 |
| I need more information about a COVID-19 vaccine’s safety and efficacy. | Disagree, Neutral, Agree | 3 |
| I am concerned a COVID-19 vaccine will have side effects. | Disagree, Neutral, Agree | 3 |
| I am concerned my insurance will not cover a COVID-19 vaccine. | Disagree, Neutral, Agree | 3 |
| I am concerned about receiving a COVID-19 vaccine because I am afraid of needles | Disagree, Neutral, Agree | 3 |
| I am comfortable taking a COVID-19 vaccine that has short term side effects such as prolonged injection site pain (e.g., redness or swelling) if the vaccine efficiently prevents COVID-19. | Disagree, Neutral, Agree | 3 |
| I am comfortable taking a COVID-19 vaccine that has short term side effects such as moderate fever (>38 degrees celsius or > 100 degrees Fahrenheit) if the vaccine efficiently prevents COVID-19. | Disagree, Neutral, Agree | 3 |
| I am comfortable taking a COVID-19 vaccine that has short term side effects such as stomach pain or nausea if the vaccine efficiently prevents COVID-19. | Disagree, Neutral, Agree | 3 |
| I would talk to my healthcare provider when considering a COVID-19 vaccine, before deciding whether or not to receive the vaccine. | 1 = Yes, 0=No | 1 |
| I would talk to none of the above when considering a COVID-19 vaccine, before deciding whether or not to receive the vaccine. | 1 = Yes, 0=No | 1 |
| I would do online searches (Google, the vaccine manufacturers website, etc) to help me decide whether or not to receive a COVID-19 vaccine. | 1 = Yes, 0=No | 1 |
| I would do not look for information to help me decide whether or  not to receive a COVID-19 vaccine | 1 = Yes, 0=No | 1 |
| Total | | 85 |
