# Supplementary material for: Factors indicating intention to vaccinate with a COVID-19 vaccine among older U.S. adults
Source: PLoS One. 2021 May 24;16(5):e0251963. doi: 10.1371/journal.pone.0251963 (PMC8143399; doi:10.1371/journal.pone.0251963)
Supplement: S3 Table — (DOCX) [file pone.0251963.s005.docx]

|  |  | **Women**  **Willing to be Vaccinated** | | | **Men**  **Willing to be Vaccinated** | | |
| --- | --- | --- | --- | --- | --- | --- | --- |
| **Question** | **Answer** | **No (n=414)** | **Yes (n=3009)** | **No (n=228)** | | **Yes (n=3751)** |  |
| In your opinion, how important are vaccines in general? | Not important at all | 8 (1.9%) | 0 (0%) | 6 (2.6%) | | 0 (0%) |  |
|  | Not very important | 26 (6.3%) | 8 (0.3%) | 17 (7.5%) | | 9 (0.2%) |  |
|  | Somewhat important | 144 (34.8%) | 120 (4.0%) | 88 (38.6%) | | 180 (4.8%) |  |
|  | Very important | 236 (57.0%) | 2881 (95.7%) | 117 (51.3%) | | 3562 (95.0%) |  |
| In your opinion, how safe are vaccines in general? | Not at all safe | 10 (2.4%) | 1 (0.0%) | 7 (3.1%) | | 0 (0%) |  |
|  | Not very safe | 37 (8.9%) | 9 (0.3%) | 21 (9.2%) | | 8 (0.2%) |  |
|  | Somewhat safe | 219 (52.9%) | 526 (17.5%) | 123 (53.9%) | | 575 (15.3%) |  |
|  | Very safe | 148 (35.7%) | 2473 (82.2%) | 77 (33.8%) | | 3168 (84.5%) |  |
| Once available to the public, how willing would you be to receive a vaccine to protect you against COVID-19? | Not at all willing | 134 (32.4%) | - | 62 (27.2%) | | - |  |
|  | Not very willing | 280 (67.6%) | - | 166 (72.8%) | | - |  |
|  | Somewhat willing | - | 1134 (37.7%) | - | | 921 (24.6%) |  |
|  | Very Willing | - | 1875 (62.3%) | - | | 2830 (75.4%) |  |
| COVID-19 is highly contagious. | Disagree | 15 (3.6%) | 6 (0.2%) | 23 (10.1%) | | 18 (0.5%) |  |
|  | Neutral | 75 (18.1%) | 119 (4.0%) | 53 (23.2%) | | 209 (5.6%) |  |
|  | Agree | 324 (78.3%) | 2884 (95.8%) | 152 (66.7%) | | 3524 (93.9%) |  |
| COVID-19 is highly severe. | Disagree | 50 (12.1%) | 75 (2.5%) | 52 (22.8%) | | 135 (3.6%) |  |
|  | Neutral | 118 (28.5%) | 501 (16.7%) | 60 (26.3%) | | 716 (19.1%) |  |
|  | Agree | 246 (59.4%) | 2433 (80.9%) | 116 (50.9%) | | 2900 (77.3%) |  |
| I believe I am at risk of getting COVID-19. | Disagree | 77 (18.6%) | 143 (4.8%) | 48 (21.1%) | | 137 (3.7%) |  |
|  | Neutral | 140 (33.8%) | 733 (24.4%) | 76 (33.3%) | | 826 (22.0%) |  |
|  | Agree | 197 (47.6%) | 2133 (70.9%) | 104 (45.6%) | | 2788 (74.3%) |  |
| Once approved, I believe a COVID-19 vaccine would be safe and effective. | Disagree | 129 (31.2%) | 10 (0.3%) | 69 (30.3%) | | 9 (0.2%) |  |
|  | Neutral | 270 (65.2%) | 815 (27.1%) | 144 (63.2%) | | 696 (18.6%) |  |
|  | Agree | 15 (3.6%) | 2184 (72.6%) | 15 (6.6%) | | 3046 (81.2%) |  |
| Once approved, I believe a COVID-19 vaccine will help protect myself and others. | Disagree | 80 (19.3%) | 1 (0.0%) | 53 (23.2%) | | 2 (0.1%) |  |
|  | Neutral | 304 (73.4%) | 473 (15.7%) | 153 (67.1%) | | 388 (10.3%) |  |
|  | Agree | 30 (7.2%) | 2535 (84.2%) | 22 (9.6%) | | 3361 (89.6%) |  |
| I need more information about a COVID-19 vaccine’s safety and efficacy. | Disagree | 21 (5.1%) | 162 (5.4%) | 8 (3.5%) | | 356 (9.5%) |  |
|  | Neutral | 27 (6.5%) | 441 (14.7%) | 24 (10.5%) | | 773 (20.6%) |  |
|  | Agree | 366 (88.4%) | 2406 (80.0%) | 196 (86.0%) | | 2622 (69.9%) |  |
| I am concerned a COVID-19 vaccine will have side effects. | Disagree | 4 (1.0%) | 291 (9.7%) | 2 (0.9%) | | 589 (15.7%) |  |
|  | Neutral | 52 (12.6%) | 1517 (50.4%) | 50 (21.9%) | | 2127 (56.7%) |  |
|  | Agree | 358 (86.5%) | 1201 (39.9%) | 176 (77.2%) | | 1035 (27.6%) |  |
| I am concerned my insurance will not cover a COVID-19 vaccine. | Disagree | 201 (48.6%) | 1540 (51.2%) | 122 (53.5%) | | 2177 (58.0%) |  |
|  | Neutral | 147 (35.5%) | 900 (29.9%) | 82 (36.0%) | | 1115 (29.7%) |  |
|  | Agree | 66 (15.9%) | 569 (18.9%) | 24 (10.5%) | | 459 (12.2%) |  |
| I am concerned about receiving a COVID-19 vaccine because I am afraid of needles | Disagree | 366 (88.4%) | 2842 (94.4%) | 201 (88.2%) | | 3542 (94.4%) |  |
|  | Neutral | 27 (6.5%) | 122 (4.1%) | 19 (8.3%) | | 157 (4.2%) |  |
|  | Agree | 21 (5.1%) | 45 (1.5%) | 8 (3.5%) | | 52 (1.4%) |  |
| I am comfortable taking a COVID-19 vaccine that has short term side effects such as prolonged injection site pain (e.g., redness or swelling) if the vaccine efficiently prevents COVID-19. | Disagree | 123 (29.7%) | 20 (0.7%) | 65 (28.5%) | | 19 (0.5%) |  |
|  | Neutral | 113 (27.3%) | 139 (4.6%) | 78 (34.2%) | | 210 (5.6%) |  |
|  | Agree | 178 (43.0%) | 2850 (94.7%) | 85 (37.3%) | | 3522 (93.9%) |  |
| I am comfortable taking a COVID-19 vaccine that has short term side effects such as moderate fever (>38 degrees celsius or > 100 degrees Fahrenheit) if the vaccine efficiently prevents COVID-19. | Disagree | 162 (39.1%) | 96 (3.2%) | 89 (39.0%) | | 101 (2.7%) |  |
|  | Neutral | 138 (33.3%) | 415 (13.8%) | 74 (32.5%) | | 486 (13.0%) |  |
|  | Agree | 114 (27.5%) | 2498 (83.0%) | 65 (28.5%) | | 3164 (84.4%) |  |
| I am comfortable taking a COVID-19 vaccine that has short term side effects such as stomach pain or nausea if the vaccine efficiently prevents COVID-19. | Disagree | 240 (58.0%) | 430 (14.3%) | 119 (52.2%) | | 318 (8.5%) |  |
|  | Neutral | 122 (29.5%) | 847 (28.1%) | 66 (28.9%) | | 894 (23.8%) |  |
|  | Agree | 52 (12.6%) | 1732 (57.6%) | 43 (18.9%) | | 2539 (67.7%) |  |
| I would talk to my healthcare provider when considering a COVID-19 vaccine, before deciding whether or not to receive the vaccine. | | 283 (68.4%) | 2750 (91.4%) | 142 (62.3%) | | 3335 (88.9%) |  |
| I would talk to none of the above when considering a COVID-19 vaccine, before deciding whether or not to receive the vaccine. | | 70 (16.9%) | 100 (3.3%) | 45 (19.7%) | | 200 (5.3%) |  |
| I would do online searches (Google, the vaccine manufacturers website, etc) to help me decide whether or not to receive a COVID-19 vaccine. | | 177 (42.8%) | 1467 (48.8%) | 83 (36.4%) | | 1845 (49.2%) |  |
| I would not look for information to help me decide whether or not to receive a COVID-19 vaccine | | 71 (17.1%) | 76 (2.5%) | 44 (19.3%) | | 128 (3.4%) |  |
