# Supplementary material for: Factors indicating intention to vaccinate with a COVID-19 vaccine among older U.S. adults
Source: PLoS One. 2021 May 24;16(5):e0251963. doi: 10.1371/journal.pone.0251963 (PMC8143399; doi:10.1371/journal.pone.0251963)
Supplement: S4 Table — November 9, 2020, Pfizer announced a first interim analysis reporting >90% efficacy of their vaccine candidate. The impact of this news on willingness was evaluated among those who answered the survey before the 9th to those who answered thereafter (excluding those who answered on the 9th itself). Those after November 9th were more willing to vaccinate (odds ratio 1.41, 95%CI 1.21–1.65). (DOCX) [file pone.0251963.s006.docx]

| **Gender** | **Time period** | **Not at all willing** | **Not very willing** | **Somewhat willing** | **Very Willing** | **Total** |
| --- | --- | --- | --- | --- | --- | --- |
| Women | Before November 9^th^ | 109 (4.2%) | 224 (8.6%) | 902 (34.5%) | 1377 (52.7%) | 2612 |
|  | After November 9th | 7 (1.9%) | 30 (8.0%) | 112 (29.7%) | 228 (60.5%) | 377 |
| Men | Before November 9^th^ | 51 (1.7%) | 136 (4.4%) | 757 (24.7%) | 2116 (69.2%) | 3060 |
|  | After November 9th | 5 (1.1%) | 19 (4.1%) | 83 (18.1%) | 351 (76.6%) | 458 |
| **Overall** | Before November 9^th^ | 160 (2.8%) | 360 (6.3%) | 1659 (29.2%) | 3493 (61.6%) | 5672 |
|  | After November 9th | 12 (1.4%) | 49 (5.9%) | 195 (23.4%) | 579 (69.3%) | 835 |
